# Comparative genomics of *Riemerella anatipestifer* reveals genetic diversity

**DOI:** 10.1186/1471-2164-15-479

**Published:** 2014-06-17

**Authors:** Xiaojia Wang, Wenbin Liu, Dekang Zhu, LinFeng Yang, MaFeng Liu, Sanjun Yin, MingShu Wang, RenYong Jia, Shun Chen, KunFeng Sun, Anchun Cheng, Xiaoyue Chen

**Affiliations:** Avian Disease Research Center, College of Veterinary Medicine of Sichuan Agricultural University, 46# Xinkang Road, Ya’an, Sichuan 625014 P.R. China; BGI-Shenzhen, Shenzhen, 518083 China; Institute of Preventive Veterinary Medicine, Sichuan Agricultural University, Chengdu, Sichuan 611130 P.R. China; Key Laboratory of Animal Disease and Human Health of Sichuan Province, Sichuan Agricultural University, Chengdu, Sichuan 611130 P. R. China

**Keywords:** *Riemerella anatipestifer*, Comparative genomics, Structural variation

## Abstract

**Background:**

*Riemerella anatipestifer* is one of the most important pathogens of ducks. However, the molecular mechanisms of *R. anatipestifer* infection are poorly understood. In particular, the lack of genomic information from a variety of *R. anatipestifer* strains has proved severely limiting.

**Results:**

In this study, we present the complete genomes of two *R. anatipestifer* strains, RA-CH-1 (2,309,519 bp, Genbank accession CP003787) and RA-CH-2 (2,166,321 bp, Genbank accession CP004020). Both strains are from isolates taken from two different sick ducks in the SiChuang province of China. A comparative genomics approach was used to identify similarities and key differences between RA-CH-1 and RA-CH-2 and the previously sequenced strain RA-GD, a clinical isolate from GuangDong, China, and ATCC11845.

**Conclusion:**

The genomes of RA-CH-2 and RA-GD were extremely similar, while RA-CH-1 was significantly different than ATCC11845. RA-CH-1 is 140,000 bp larger than the three other strains and has 16 unique gene families. Evolutionary analysis shows that RA-CH-1 and RA-CH-2 are closed and in a branch with ATCC11845, while RA-GD is located in another branch. Additionally, the detection of several iron/heme-transport related proteins and motility mechanisms will be useful in elucidating factors important in pathogenicity. This information will allow a better understanding of the phenotype of different *R. anatipestifer* strains and molecular mechanisms of infection.

## Background

*Riemerella anatipestifer* (RA) is a Gram-negative bacterium in the family Flavobacteriaceae and rRNA superfamily V [1]. *R. anatipestifer* can infect ducks, geese, turkeys, chickens, and other birds, and leads to a contagious septicemia [2]. Transmission between ducks occurs vertically (through the egg) as well as horizontally via the respiratory tract [3]. *R. anatipestifer* has a worldwide distribution and is one of the leading problems of the farmed duck industry, mainly infecting young ducks with a mortality of up to 90%. Animals that survive infection may be stunted [4], leading to decreased production. Riemerellosis causes substantial economic losses in countries with significant duck industries, such as China and Southeastern Asia [5]. While serotyping is the traditional method to differentiate *R. anatipestifer* isolates [5], other methods, including PCR based on 16S rRNA or *rpoB* genes [6, 7], repetitive-sequence polymerase chain reaction (Rep-PCR) [8], multiplex PCR [9], matrix-assisted laser desorption/ionization-time of flight (MALDI-TOF) mass spectrometry [10], plasmid profiling, pulsed-field gel electrophoresis (PFGE), and PCR-restriction fragment length polymorphism (PCR-RFLP) [11] have also been used to characterize isolates.

At least 21 serotypes have been described in different countries [5, 7, 12] with no cross-protection between different serotypes. Among pathogenic isolates, serotypes 1, 2, 3, 5, and 15 are the most common [13]. Individual animals can be infected with multiple serotypes and changes in the predominant serotype from year to year within a single farm have been described [12].

Although Reimerellosis causes serious economic losses, the pathogenesis of *R. anatipestifer* and the virulence factors remain mostly unknown. Subramaniam et al. identified OmpA as a predominant immunogenic outer membrane protein [14]. Later, it was shown that *ompA* mutant strains were attenuated when used to infect ducklings, with decreased adhesion and invasion capacities in Vero cells, indicating that OmpA is a virulence factor [15]. Recently, Zhai et al. selected six proteins that cross-reacted with serotypes 1 and 2 for a vaccine trial. Only administration of the recombinant outer membrane protein A (OmpA) showed a protective effect when challenged by serotype 1 (60%) and serotype 2 (50%) [13]. Additionally, VapD was identified as a virulence factor, with homology to virulence-associated proteins of other bacteria [16]. CAMP cohemolysin was identified as another potential virulence factor, which may lyse red blood cells and release iron for use by the organism [17].

The publication of the first *R. anatipestifer* genome, ATCC11845 [18] has improved the understanding of the disease mechanisms underlying infection. However, the relatively limited number of published strains has hindered more in-depth analysis. In order to establish genetic differences between pathogenic strains, we sequenced two *R. anatipestifer* genomes, RA-CH-1 and RA-CH-2, which were isolated from sick ducks from Chengdu and Mianyang, respectively, in the SiChuan province of China, and compared these to the two previously sequenced strains, ATCC11845 and RA-GD (which was isolated from a sick duck in GuangDong, China).

## Results and discussion

### General features of the *R. anatipestifer*genomes

Genomic read-data for the two *R. anatipestifer* strains sequenced in this study were generated using a multiplexing approach in a single Illumina HiSeq lane. The resulting sequences were assembled using SOAPdenovo. The previously sequenced ATCC11845 has single 2,164,087 bp circular chromosome with 35.01% GC content. In contrast, RA-CH-1 is larger at 2,309,519 bp with 35.07% GC content while RA-CH-2 is similar at 2,166,321 bp with 35.04% GC content. ATCC11845, RA-CH-1 and RA-CH-2 contain 2,091 (92.8% of the genome), 2,236 (97.8%), and 2,095 (97.8%) genes, respectively. All genomes have approximately the same codon usage frequency.

### Genes associated with iron/hemin metabolism

Bacteria that reside in animal tissues must acquire iron from their host for growth. A large number of genes coding for iron and hemin metabolism and iron-dependent transcriptional regulators were annotated in all sequenced strains. A total of one siderophore-interacting protein (Sip) and three siderophore receptors were detected that could be involved in Fe^3+^ uptake. The siderophore-interacting protein was previously found to be involved in iron utilization and mutation of this gene significantly decreased virulence in *R. anatipestifer* CH-3 [19]. There were two putative proteins, FeoA and FeoB, for Fe^2+^ uptake and two outer membrane hemin receptors. All sequenced strains had one extracellular hemin-binding protein (hemophore), and no hemin degrading proteins were detected via sequence analysis, suggesting that *R. anatipestifer* may have a novel hemin degrading system. Additionally, we found that several TonB-dependent receptors with a plug domain, one set of the ExbB-ExbD-TonB complex, one set of the ExbB-ExbD-ExbD-TonB complex, and one TonB family protein. The TonB-dependent receptor TbdR1 (Riean_1607) has been found to be involved in heme acquisition in *R. anatipestifer*. The median lethal dose of a *tbdR1* mutant was approximately 45-fold higher than the wild-type CH-3 strain [20]. Our group has confirmed the functions of TonB and the TonB complex and determined that the ExbB-ExbD-TonB complex is involved in heme uptake in ATCC11845 (unpublished data).

*R. anatipestifer* is usually grown on blood-enriched media. Sequence analysis shows that *R. anatipestifer* does not encode for genes involved in heme synthesis, *hemF, Y,* and *G* (http://www.kegg.jp/pathway/rae00860). This suggests that the heme compounds from the culture plate could be essential for growth*.* We have determined that *R. anatipestifer* can synthesize hemin using protoporphyrin as a substrate and subsequently use hemin as an iron source (unpublished data). However, the function of proteins involved in iron/hemin metabolism in maintaining and enhancing virulence still requires experimental investigation.

### Genes associated with gliding motility

Cells of the phylum Bacteroidetes can rapidly move over surfaces using a process called gliding motility. In *F. johnsoniae,* at least nineteen genes (*gldA*, *gldB*, *gldD*, *gldF*, *gldG*, *gldH*, *gldI*, *gldJ*, *gldK*, *gldL*, *gldM*, *gldN*, *sprA*, *sprB, sprC, sprD, sprE, sprT* and *RemA*) involved in gliding motility have been identified [21–23]. These motility proteins constitute a novel protein secretion system, the Por secretion system (PorSS) [24], which may be an integral part of the gliding motility machinery [23]. In *F. johnsoniae,* the Por secretion system consists of *gldK*, *gldL*, *gldM*, *gldN*, *sprA*, *sprE*, and *sprT*, which are needed for secretion of an extracellular chitinase [23]. Similarly, the *P. gingivalis* PorSS is needed for secretion of gingipain protease virulence factors [25].

Genome analysis finds that *R. anatipestifer* encodes for several genes involved in gliding motility, including *gldA, gldB, gldC, gldD, gldF, gldH, gldJ, gldK, gldL, gldM, gldN, porP*, and *porT*. Many of the proteins encoded by these genes are predicted to localize to the cellular envelope. In *F. johnsoniae, gldK, gldL, gldM,* and *gldN* are clustered together on in two adjacent operons, although *gldK* is transcribed separately from the other three genes [26]. A similar arrangement is found in *R. anatipestifer* as well as other *Bacteroidetes,* such as *F. psychrophilum* and *C. hutchinsonii* [21]. This organization suggests that the protein products of these genes work together as a part of a complex, and the extensive conservation of the genes encoding this protein secretion system indicates it is likely functional in *R. anatipestifer.* Analysis of this system in *R. anatipestifer* has the potential to provide insight into disease pathogenesis. For example, in *P. gingivalis,* the PorSS is involved in gliding motility and pathogenesis [24]. The PorSS, its relationship to gliding, and its function in pathogenesis, needs to be further studied in *R. anatipestifer*.

### Complete genome analysis and structural variation

**Figure 1:**
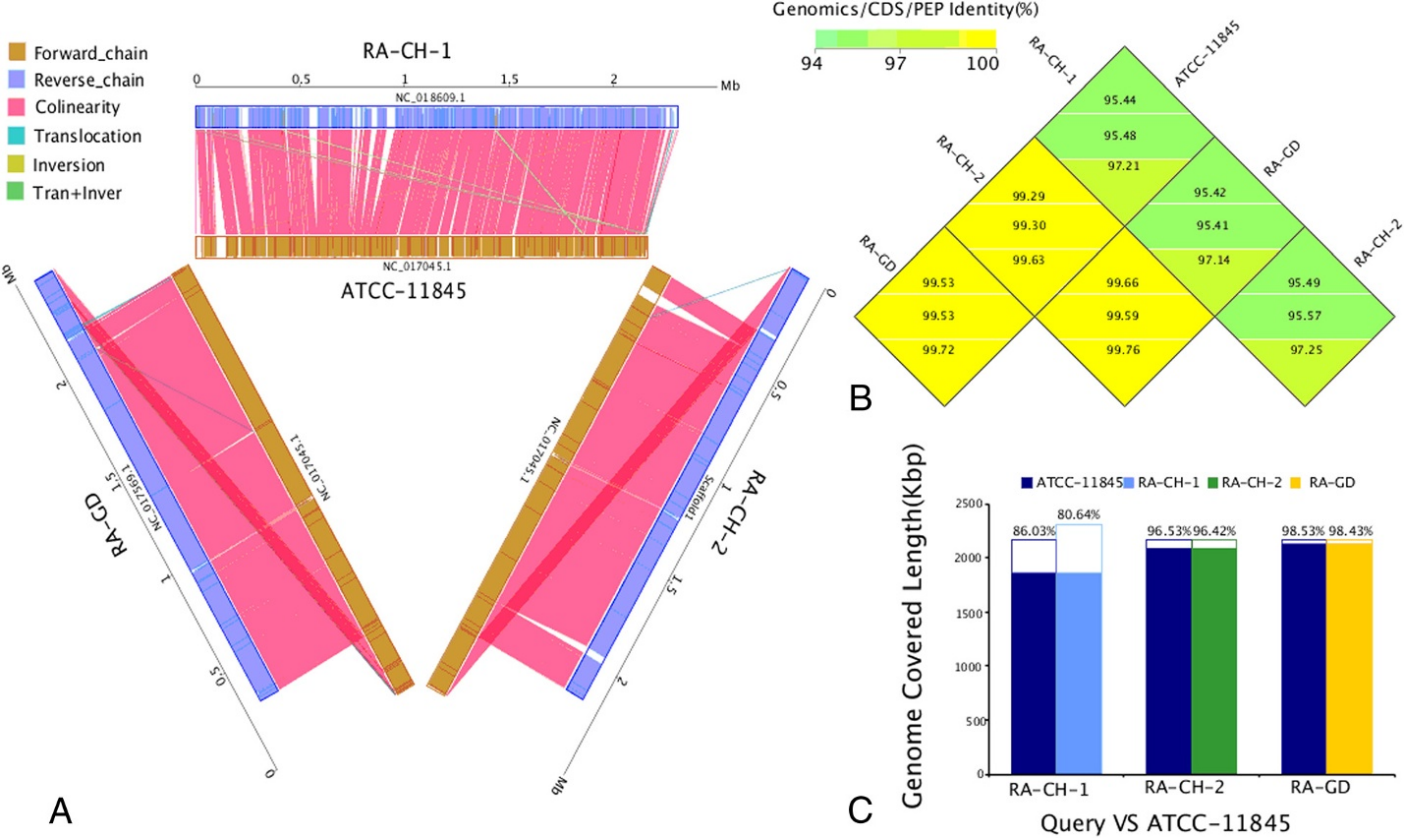
**Whole-genome collinearity comparison. A:** Collinearity comparison results. All three strains have deleted sequences (blanks) compared to ATCC11845. **B:** Genome-wide colinear homology comparision. From top to bottom: genome similarity, coding sequence (CDS) similarity, and predicted amino acid sequence similarity. **C:** Coverage statistics.

**Figure 2:**
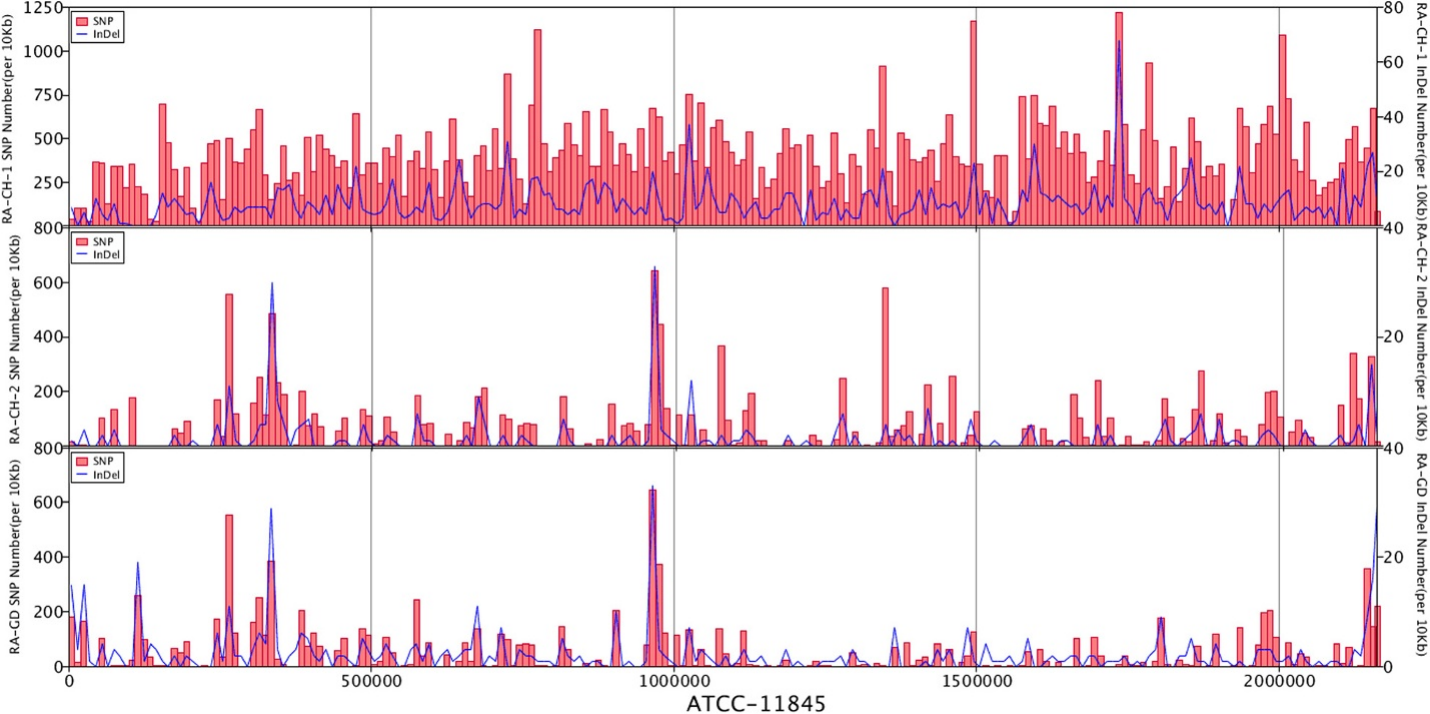
**Genome SNP-indel maps.** The distribution of SNP and indels in RA-CH-1, RA-CH-2 and RA-GD. SNP distribution is presented as a bar graph and indel distribution as a line graph.

**Figure 3:**
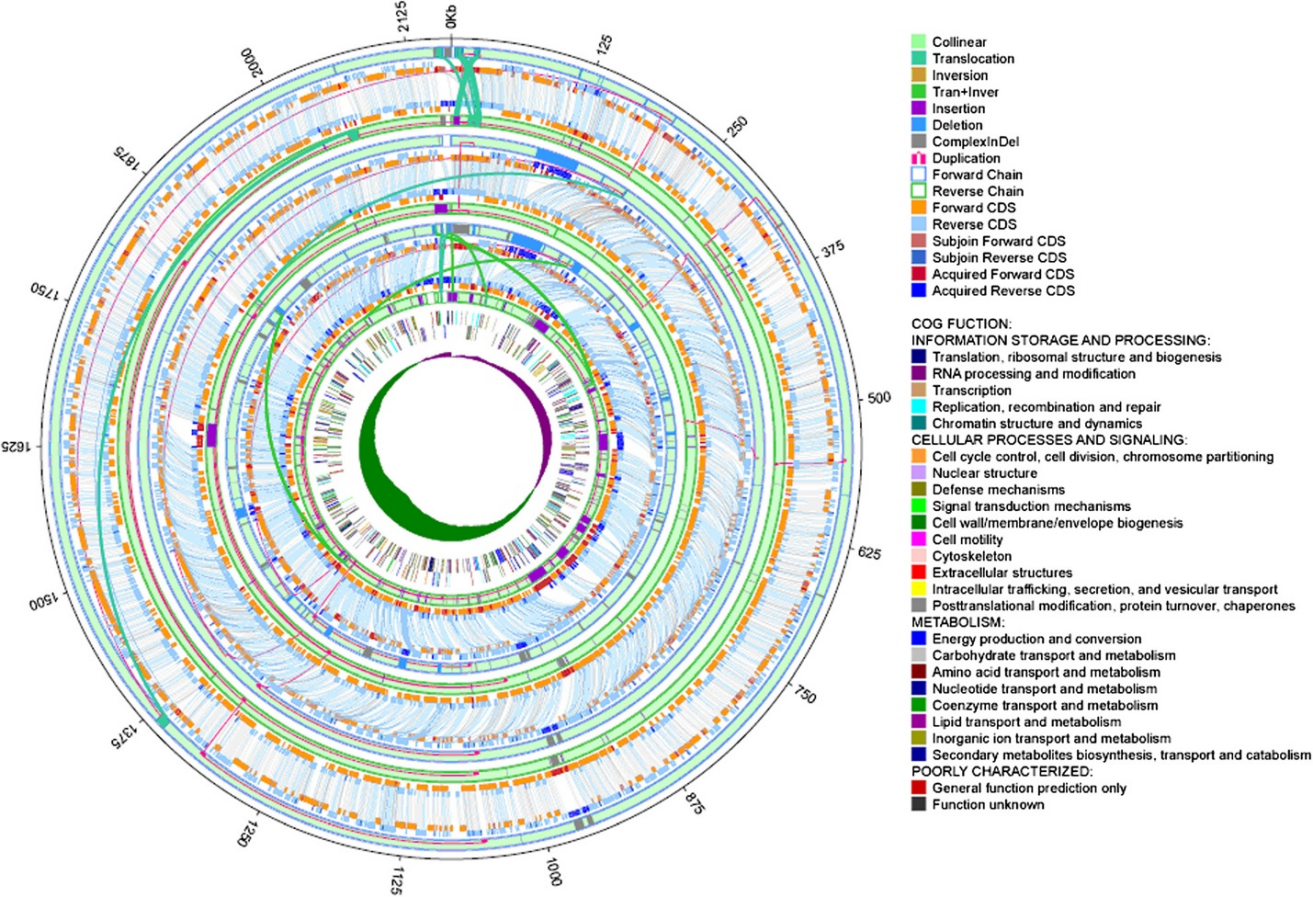
**Genome structure variation and gene pairing.** From inside to outside: GC-skew of ATCC11845, the COG functional assignments of ATCC11845, structural variation of RA-CH-1, RA-CH-2, and RA-GD compared to ATCC11845. Purple is positive, green is negative.

The genomes of RA-CH-2 and RA-GD were similar to the genome of ATCC11845, while the genome of RA-CH-1 is significantly different. All four strains had some deletions unique to a specific strain. The missing parts of the RA-CH-2 and RA-GD genomes were focused in three different places as shown in the colinearity analysis (Figure 1A). However, deleted sequences of RA-CH-1 were dispersed throughout the genome (Figure 1A). Moreover, there were more genome rearrangements between RA-CH-1 and ATCC11845 than the other two genomes. By analyzing the genomic coverage rate and sequence similarity of homologous regions for the four different genomes, we found that there is a higher degree of similarity between ATCC11845 and both RA-CH-2 and RA-GD than RA-CH-1 (Figures 1B and C). Compared to the genome of ATCC11845, RA-CH-1 had a higher SNP and indel density than RA-CH-2 and RA-GD. The distribution of SNPs and indels for RA-CH-2 and RA-GD are similar (Figure 2). Furthermore, we found that the genomes of RA-CH-1 and RA-CH-2 had commons deletions compared to ATCC11845 and RA-GD (Figure 3). Both RA-CH-1 and RA-CH-2 contain same inserted and deleted sequences, which suggests these deletions are localized to the region both these strains were isolated from.

### Functional analysis of variant genes

**Figure 4:**
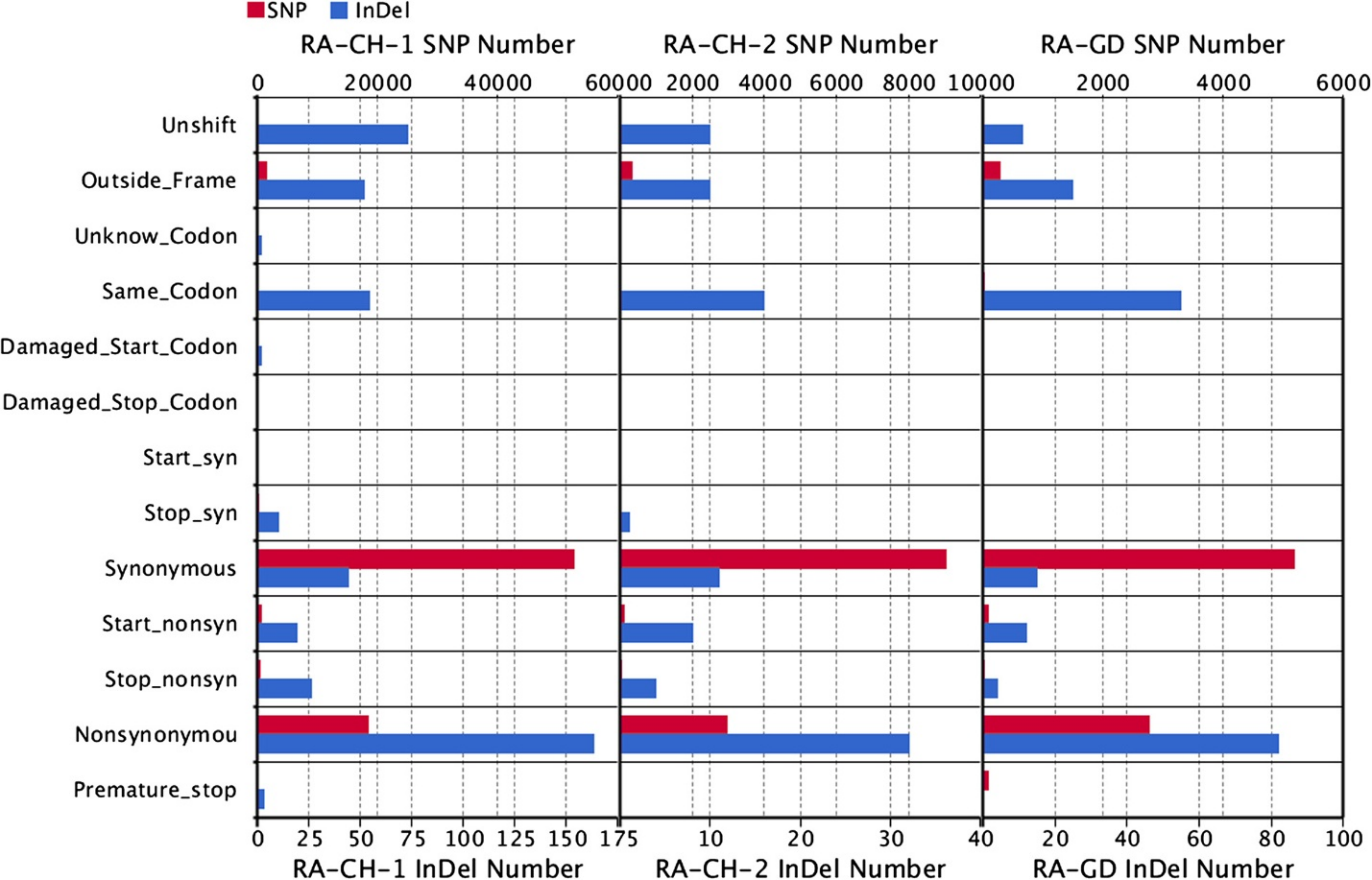
**Variations due to SNPs and indels.** The number of SNPs is in red, and the number of indels in blue. Unshift: the mutation does not cause a frame change (only for indels), Outside_Frame: the variation occurred outside the coding frame, Unknow_Codon: unrecognized variant codon, Same_Codon: the codon was unchanged by the mutation, Start_nonsyn: non-synonymous mutations at start codon; Stop_nonsyn: non-synonymous mutations at stop codon; Start_syn: synonymous mutations at start codon; Stop_syn: synonymous mutations at stop codon, Total_Mutate: the total number of various variants.

**Figure 5:**
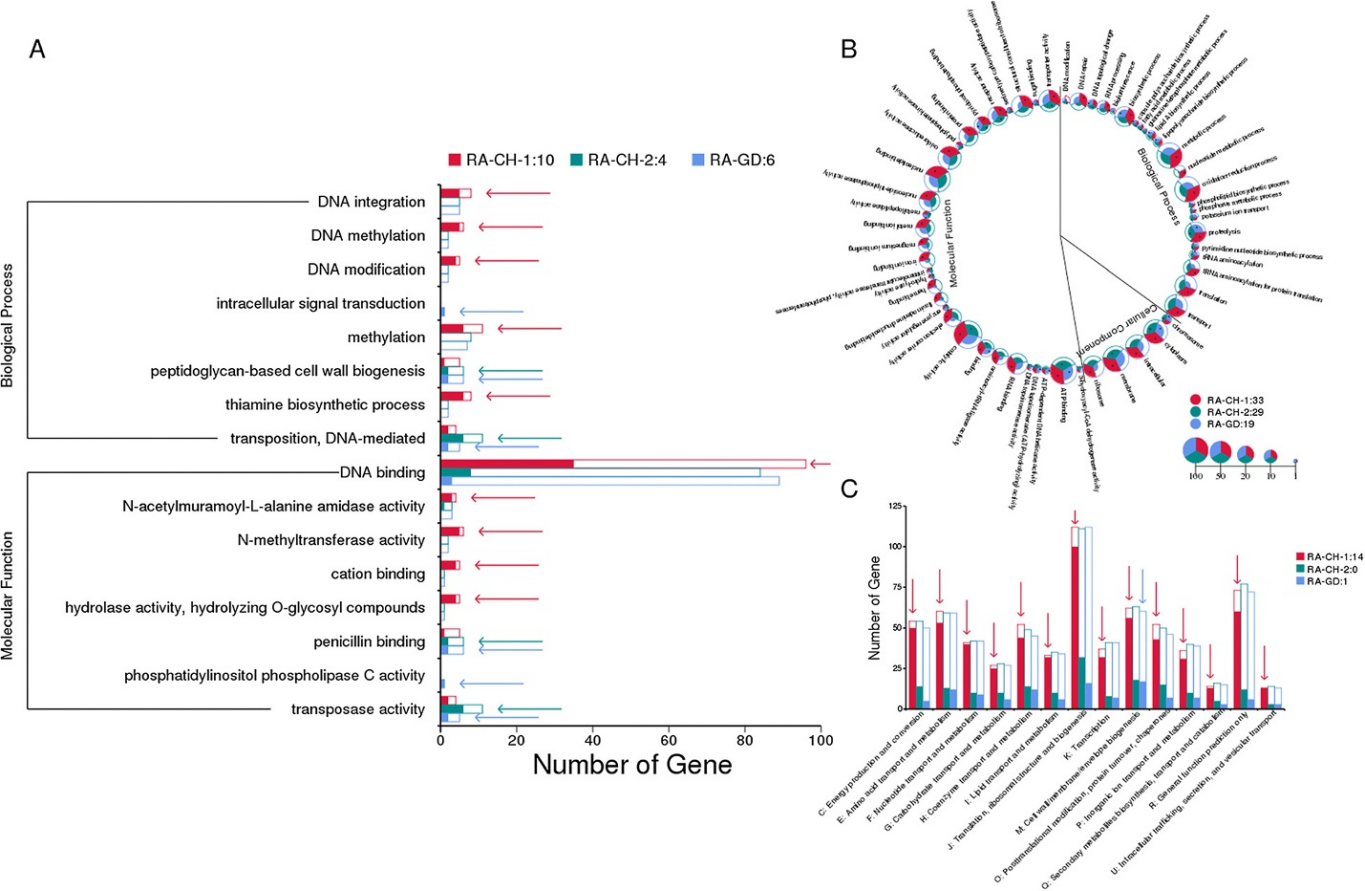
**Correlation of variations with functional enrichment analysis. A:** Functional characterization of insert region genes using the GO database; the arrows show significant enrichment. **B:** Functional characterization of genes containing SNPs, and indels using the GO database; asterisks show significant enrichment. **C:** Functional characterization of genes containing SNPs, and indels using the COG database; arrows show significant enrichment.

Based on analysis of mutation types, we found that indels mainly induced non-synonymous mutations, while SNP primarily caused synonymous mutations. There were significantly more SNPs than indels (Figure 4). In addition, we analyzed three different types of genes using COG [27], KEGG [28], PATHWAY [29], and GO functional characterization databases [30] (Figure 5). First were genes that were found by pairing with sequences from other strains, but were not annotated because of a mutation in the original sequence. Second were structural variations (SV) region genes. Third were genes containing SNPs or small indels. Among these three groups, the first have no significant difference in the three databases, indicating that there is no increased rates of change in any particular functional gene category or pathway.

Through COG analysis, we can find that the SV region sequences of RA-CH-1, RA-CH-2, RA-GD have no significant differences in polymorphism frequencies. RA-CH-1 had 14 areas with significantly higher rates of SNPs and indels, RA-CH-2 showed no significant differences, and RA-GD had significantly higher SNP and indel rates in the COG “M: Cell wall/membrane/envelope biogenesis”, which may be associated with host invasion or antibiotic resistance. SNPs are the main type of polymorphism, indicating that *R. anatipestifer* evolution mainly relies on this rather than deletion or insertion to generate genetic diversity.

### Gene family cluster analysis

**Figure 6:**
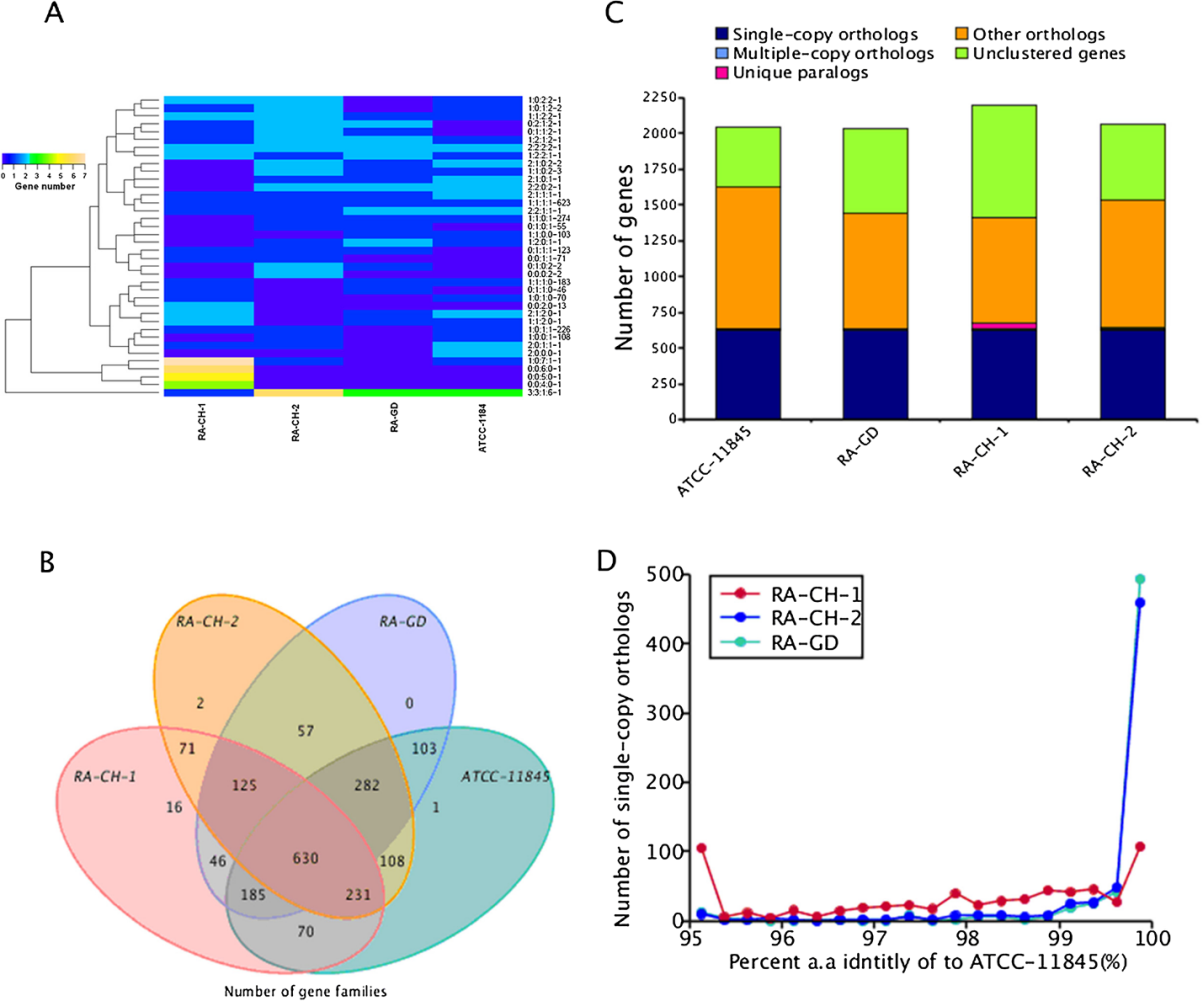
**Gene family analysis. A.** Distribution of gene family member copy numbers. **B.** Venn diagram of homologous gene families. **C.** The number of homologous genes among four *R. anatipestifer* genomes. **D.** Single-copy homologous gene similarity distribution.

A gene family is a set of several similar genes formed by duplication of a single original gene, generally with similar biochemical functions [31]. Members of the same gene family can be closely arranged, forming a gene cluster, or can be scattered throughout the chromosome, with different patterns of expression and regulation. Gene families play an important role in the evolution and functional analysis of different species [32]. For *R. anatipestifer*, most of the high copy number gene families were annotated as hypothetical genes, with RA-CH-2 having a higher copy number compared to the other strains (Figure 6A). The number of gene families in different strains reflected phenotypic differences. Copy numbers of core gene families may be related to quantitative traits, while non-core gene families may be associated with strain-specific traits. RA-CH-1 had four non-core gene families with higher copy numbers, but these were not detected using the KEGG or COG databases. Multi-copy gene family analysis showed that the four strains analyzed had only six multi-copy gene families, with RA-CH-2 family members having higher copy numbers. Overall, RA-CH-1 had the most unique family members (up to 16), while the other three strains had only 1–2 unique gene families per strain (Figure 6B). RA-CH-1 had 787 unique genes, while each of the other three strains had approximately 500 unique genes each. This complexity could reflect the differing biological characteristics of *R. anatipestifer* strains.

### Phylogenetic analysis

**Figure 7:**
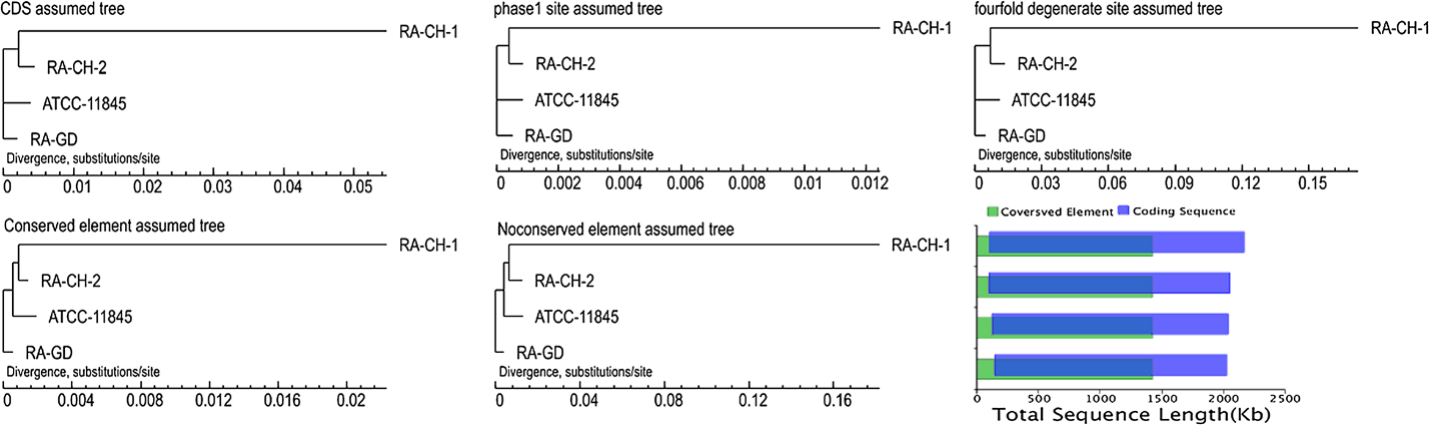
**The phylogenetic tree of four** ***Riemerella.*** The phylogenetic trees were constructed using orthologous gene coding sequences, phase 1 site, and four-fold degenerate sites, respectively. As the relationship of the ancestor node of RA-CH-1 and RA-CH-2 with ATCC11845 and RA-GD was not clear, the phylogenetic trees were constructed using conserved (top) and non-conserved (bottom) elements.

**Figure 8:**
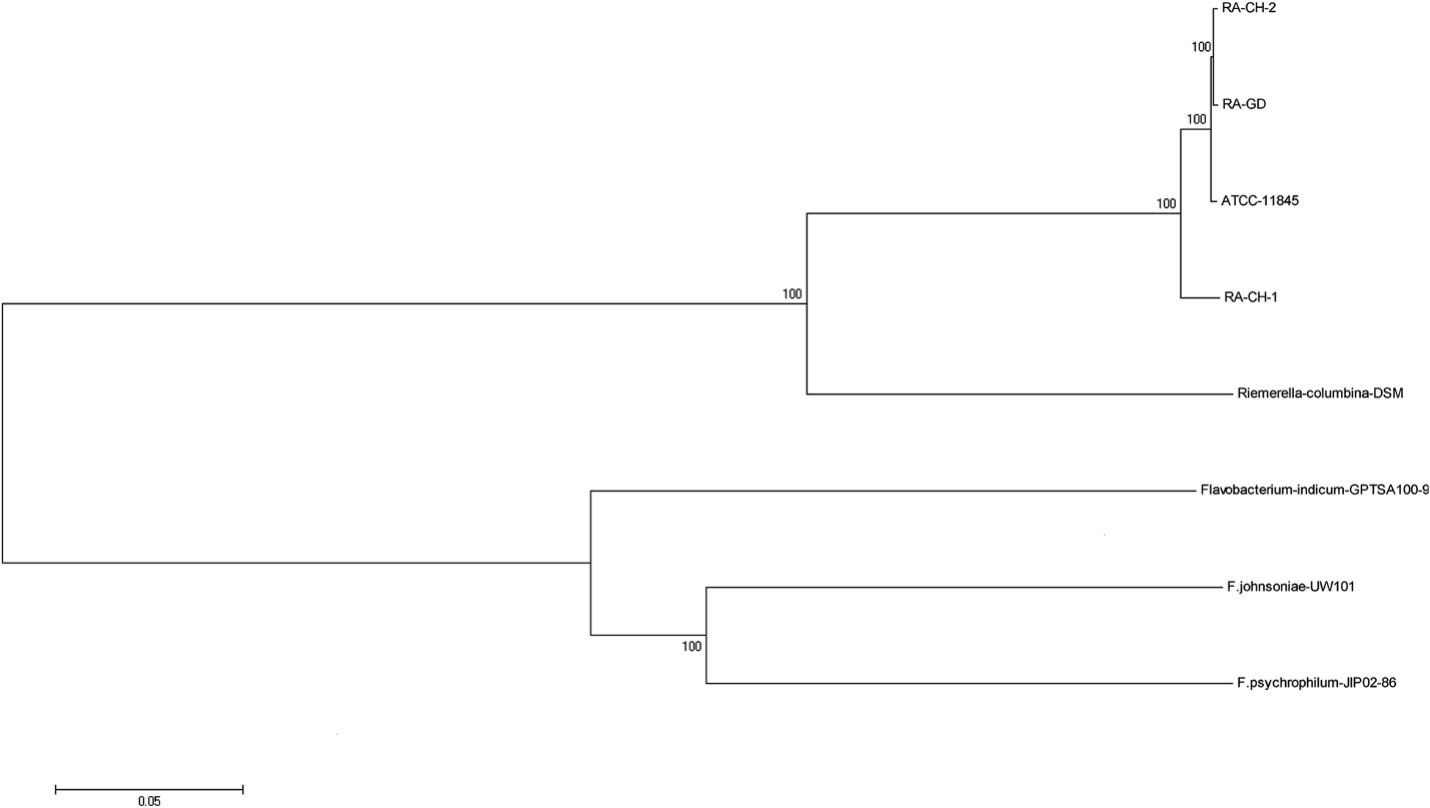
**Phylogenetic relationships between** ***Riemerella*** **and other related Flavobacteria.** Phylogenetic relationships based on maximum likelihood analysis of genome sequences. Support for monophyletic groups by bootstrap analysis is indicated as numbers out of 100. The scale bar represents sequence variation based on the models for nucleotide substitution and tree shape used in the maximum likelihood analysis.

We constructed two phylogenetic trees of the four *R. anatipestifer* strains using conservative and non-conservative elements. The topological structures of the conservative and non-conservative trees are similar, but with different branch lengths (Figure 7). Phylogenetic analysis determined that RA-CH-1 and RA-CH-2 belong to the same branch, but the relationship of their ancestor node with the ATCC11845 and RA-GD strains was unclear (Figure 7). RA-CH-1 and RA-CH-2 appear closely related to ATCC11845, but are on different branches compared to RA-GD (Figure 7). Additionally, structural analysis demonstrated that some conserved components were not in annotated CDSs. Most of the conserved components were in conserved regions and non-conserved regions had eight times the polymorphism rate of conserved regions (Figure 7). When compared to other flavobacteria, the four *R. anatipestifer* strains and *R. columbina* cluster in one group, while other flavobacterium belong to another group (Figure 8).

## Conclusions

We successfully isolated two *R. anatipestifer* strains, RA-CH-1 and RA-CH-2, from Chengdu and Mianyang, respectively, in the SiChuan province of China, and completed their genome sequences. Using a mixture of comparative genomics strategies, we completed a comprehensive analysis of four *R. anatipestifer* strains: RA-CH-1, RA-CH-2, ATCC11845, and RA-GD, to identify factors involved in pathogenesis. Our findings will form the foundation of future investigations into the pathogenesis of *R. anatipestifer.*

## Methods

### Genome sequencing and annotation

#### Specimen collection and DNA extraction

RA-CH-1 and RA-CH-2 were isolated from the brain of sick ducks from Chengdu and Mianyang, respectively, in the SiChuan province of China. Serotyping were performed for RA-CH-1 and RA-CH-2 using reference serum of National Animal Disease Center. RA-CH-1 is serotype 1, RA-CH-2 is serotype 2. The samples were lyophilized after two successive transfers of stock culture on tryptic soy agar (TSA, Difco, Detroit, USA) containing 5% defibrinated sheep blood at 37°C for 24–48 h. *R. anatipestifer* genomic DNA was extracted and purified via proteinase K treatment, multiple phenol extractions, ethanol precipitation, and spooling. Genomic DNA was checked for quality by monitoring A_260_/A_280_ ratios (DU800, Beckman Coulter, USA).

#### DNA sequencing and assembly

Bacterial strains were sequenced using an Illumina Hiseq2000 (Illumina Inc., San Diego, CA) with a multiplexed protocol. Paired-end 90 nt long reads from 500 bp and 6 kb random sequencing libraries were generated for strains RA-CH-1, and RA-CH-2. Raw data in four steps, including removing reads with 5 bp of ambiguous bases, removing reads with 20 bp of low quality (≤Q20) bases, removing adapter contamination, and removing duplicated reads. Finally, 100 × 500 bp and 50 × 6 kb libraries were obtained with clean paired-end read data. Assembly was performed using SOAPdenovo v1.05 [33] (http://soap.genomics.org.cn/soapdenovo.html). The genome of the RA-GD strain was downloaded from NCBI (ftp://ftp.ncbi.nih.gov/genbank/genomes/Bacteria/Riemerella_anatipestifer_RA_GD_uid49039).

#### Repetitive sequences analysis

The genome was searched for tandem repeats using Tandem Repeats Finder [34] and Repbase [35] to identify interspersed repeats. Transposable elements in the genome assembly were identified at both the DNA and protein levels. To identify transposable elements at the DNA level, Repeat Masker was applied using a custom library based on Repbase. For protein analysis, Repeat Protein Mask from the Repeat Masker package was used to perform RM-BlastX against a transposable elements protein database.

#### Gene predict analysis

Genes were predicted using Glimmer v3.02 [36] (http://www.cbcb.umd.edu/software/glimmer). This software predicts start sites and coding region more effectively and has better interpolation of hidden Markov models, reducing the ratio of false positive predictions.

#### Gene functional annotation

Function annotation was accomplished by analysis of protein sequences. Genes were aligned with databases to obtain the annotation corresponding to homologs, with the highest quality alignment result chosen as the gene annotation. Function annotation was completed by comparing BLAST v2.2.23 (http://blast.ncbi.nlm.nih.gov/Blast.cgi) results in M8 format to the Kyoto Encyclopedia of Genes and Genomes (KEGG) v59 [37], Cluster of Orthologous Groups of proteins (COG) v20090331 [27, 38], SwissProt v2011_10_19 [39], NR v2012-02-29, and Gene Ontology (GO) v1.419 [40] databases.

### Comparative genomic analyses

#### Structural variation

The sequences of the RA-CH-1, RA-CH-2, and RA-GD strains were compared to the reference sequence ATCC11845 using Mummer v3.22 [41] (http://mummer.sourceforge.net) for the chain stander and start side selection and LASTZ v1.01.50 [42] (http://www.bx.psu.edu/miller_lab/dist/README.lastz-1.02.00) for detailed alignment. Syntenic regions, deletions, insertions, inversions, and translocations were identified from the alignment blocks [43].

#### SNP and small indel identification

SNPs were identified in mismatch sites from syntenic regions. SNPs located in sequence gaps, repeat regions, or at scaffold ends were discarded. To validate the resulting non-redundant candidate SNPs, high-quality paired-end reads were mapped to the corresponding genomes with SOAPaligner v2.21 [44] (http://soap.genomics.org.cn), and the most abundant (n1) and the second most abundant (n2) nucleotides at each SNP position in each strain were examined. High quality SNPs were defined as those where the quality score of each mapped base was > Q20 and that satisfied the criteria n1 + n2 ≥ 10 and n1/n2 ≥ 5. If more than 95% of reads had a high-quality SNP in a certain position, the SNP was included in the final set. The resulting set of unique SNPs was filtered to obtain a set of high-quality SNPs present in all strains.

Raw small insertions and deletions (indels) were defined as those with a length shorter than 50 bp. These were identified as gaps from the synteny alignment. Any indels with more than one mismatch in the sequence 10 bp upstream and downstream of the indels were eliminated.

Read validation was performed on the remaining indels. Those with three or more reads which mapped to the indels-removed sequence of the subject were retained.

#### Phylogenetic analysis

##### Gene family analysis

Gene families were constructed using genes from ATCC11845, RA-CH-1, RA-CH-2, and RA-GD. The current analysis is aimed at single copy gene families, which are determined by aligning protein sequences via BLAST. Gene family clustering from alignment results was performed using orthomclSoftware-v2.0.3.tar.gz [45].

##### Phylogenetic tree analysis

Protein alignments were converted into multiple amino acids sequence alignments using Muscle v3.8.31 (http://www.drive5.com/muscle). Gene family trees were constructed from multiple sequences alignments using the ML method with Treebest v1.9.2 [46] (http://treesoft.svn.sourceforge.net/viewvc/treesoft/trunk/treebest).

#### Functional enrichment analysis of variant gene/proteins

Connections between all gene function variations were analyzed using the differential gene/protein function items in the COG, GO, and KEGG databases, allowing calculation of the number of corresponding COG/GO/KEGG terms. We then determined the difference between differences within each COG/GO/KEGG group and the whole genome for variant genes/proteins using the hypergeometric test [30], with a P-value ≤ 0.05 considered significant.
